# Spontaneous knee dislocation: a rare and dreadful complication of septic arthritis

**DOI:** 10.1093/jscr/rjad279

**Published:** 2023-05-22

**Authors:** Alwaleed Abdullah Alshahir, Faisal Hamad AlNaqa, Mohammed Abdulrahman Benmeakel, Khalid Abdullatif Alsheikh

**Affiliations:** Ministry of the National Guard-Health Affairs, King Abdulaziz Medical City, Riyadh, Saudi Arabia; Orthopedics Surgery Department, Ministry of the National Guard-Health Affairs, King Abdulaziz Medical City, Riyadh, Saudi Arabia; King Saud Bin Abdulaziz University for Health Sciences, Riyadh, Saudi Arabia; Orthopedics Surgery Department, Ministry of the National Guard-Health Affairs, King Abdulaziz Medical City, Riyadh, Saudi Arabia; King Saud Bin Abdulaziz University for Health Sciences, Riyadh, Saudi Arabia; King Abdullah International Medical Research Centre, Riyadh, Saudi Arabia; Orthopedics Surgery Department, Ministry of the National Guard-Health Affairs, King Abdulaziz Medical City, Riyadh, Saudi Arabia; King Saud Bin Abdulaziz University for Health Sciences, Riyadh, Saudi Arabia; King Abdullah International Medical Research Centre, Riyadh, Saudi Arabia; Orthopedics Surgery Department, Ministry of the National Guard-Health Affairs, King Abdulaziz Medical City, Riyadh, Saudi Arabia; King Saud Bin Abdulaziz University for Health Sciences, Riyadh, Saudi Arabia; King Abdullah International Medical Research Centre, Riyadh, Saudi Arabia

**Keywords:** knee dislocation, septic arthritis, arthrodesis, spontaneous dislocation

## Abstract

Spontaneous knee dislocation without a history of trauma is a rare entity to witness. Herein, we report a case of a patient who presented to the emergency department (ED) with a history of fever, chills and vomiting associated with progressive right knee swelling, pain and impaired range of motion (ROM). Physical exam of her right knee showed symmetrical swelling with diffuse tenderness and limited ROM due to pain. Joint aspirate and full septic workup confirmed the diagnosis of septic arthritis. Following her management and two events of irrigation and debridement of the septic knee, the patient was discharged. However, after 1-week from discharge, she presented to ED with right leg swelling and tenderness despite being bedbound for 3 months and denying any history of trauma with radiographs showing a posterior knee dislocation. This report aimed to shed a light on this dreadful complication of septic arthritis and highlights the importance of early recognition and management.

## INTRODUCTION

Knee dislocation is a devastating injury that can result most commonly following a high-energy trauma. However, spontaneous knee dislocation without the presence of trauma is extremely rare. Hence, the aim of this case report is to describe a patient who presented with a spontaneous knee dislocation following septic arthritis and highlights the importance of early recognition and management of this rare but potentially dreadful complication.

## CASE HISTORY

### First presentation

A 61 year old female with history of diabetes mellitus (DM) and chronic kidney disease (CKD) presented to the emergency department (ED) with 3 days history of fever, chills and vomiting associated with progressive right knee swelling, pain and impaired range of motion (ROM). Physical exam of her right knee showed symmetrical swelling with diffuse tenderness and limited ROM due to pain. Her joint aspirate and full septic workup confirmed the diagnosis of septic arthritis with bacteremia along with acute kidney injury, metabolic acidosis and respiratory failure; all secondary to sepsis. The synovial fluid and blood cultures came back positive with *Streptococcus pneumoniae*, sensitive to the empirical antibiotic that was used. The patient then was shifted to IV meropenem and vancomycin and was scheduled for surgery whenever fit for it. On the second day of her admission, the patient started to stabilize and was pushed for the operating room (OR) for irrigation and debridement (I&D) of the septic knee. Subsequently, the patient tolerated the procedure well and was transferred under the intensive care unit (ICU) and internal medicine (IM) care for her other active medical issues.

On her post-operative Day 42, the wound was noted to be complicated be dehiscence and pus discharge and another I&D was carried out. The patient was discharged on post-operative Day 20 with a 3 month course of ceftriaxone.

### Second presentation

**Figure 1:**
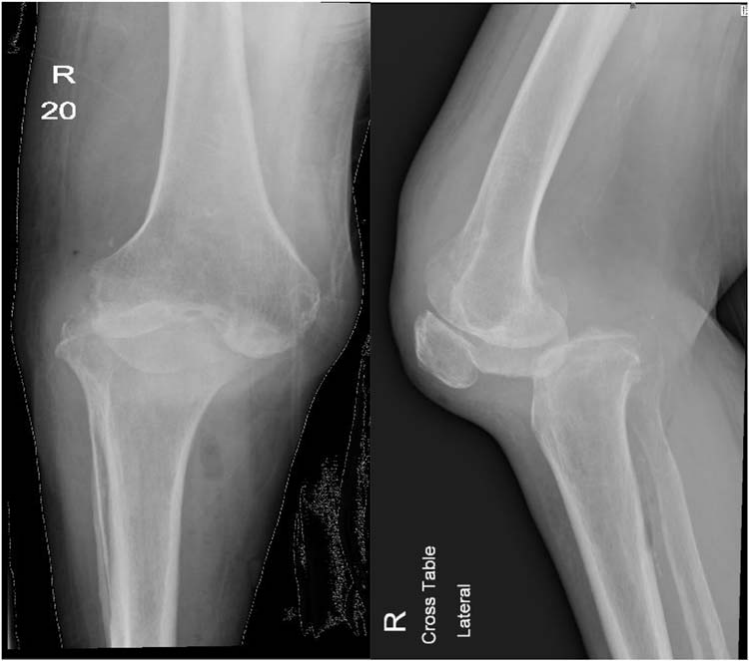
Anteroposterior and lateral views of the knee at second presentation.

After 1-week from discharge, she presented to ED with 2 days history of right leg swelling and tenderness despite being bedbound for 3 months and denying any history of trauma. Her physical exam showed right knee swelling with absent posterior tibial artery pulse. Because of her mobility status, there was a suspicion of deep vein thrombosis (DVT); therefore, doppler ultrasound was obtained along with knee X-ray and computed tomography (CT) runoff (Fig. 1). Her doppler ultrasound showed negative results; however, knee X-ray showed posterolateral knee dislocation and CT runoff revealed evidence of septic arthritis of right knee with large intramuscular abscess extending from the popliteal fossa down to above the ankle joint adjacent to the Achilles tendon. The patient then was shifted to OR for a third I&D and a spanning external fixator application to allow for soft tissue to form enough fibrosis in order to keep the joint stable and avoid further major surgeries. At her 6-week follow-up, the external fixator was removed and the patient’s assessment showed stable knee joint and unremarkable, pre- and post-external fixator removal and X-ray.

### Third presentation

**Figure 2:**
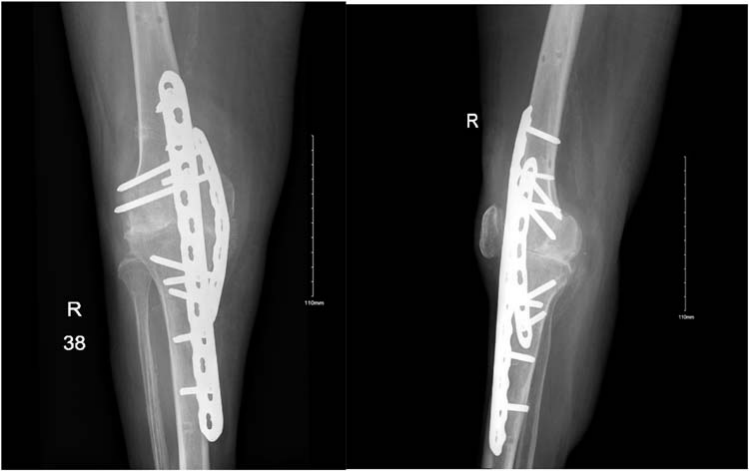
Anteroposterior and lateral views of the knee following arthrodesis.

Three days following her external fixator removal, she presented to the ED with a history of severe knee pain and a loud clicking sound after trying to ambulate. Her X-ray showed a recurrent knee dislocation. The patient was then admitted and booked for a prophylactic I&D, which showed no signs of septic arthritis and later underwent an arthrodesis of the right knee (Fig. 2). The decision of arthrodesis was made after clearing out infection, and to eliminate further dislocation concerns.

## DISCUSSION

Although spontaneous joint dislocation is a known severe complication following septic arthritis, the occurrence of such sequelae is rarely seen and reported. Through the literature study, we were able to identify two cases only of spontaneous knee dislocation due to septic arthritis (Table 1).

**Table 1:** A review of the literature on spontaneous knee dislocation following septic arthritis

First author	Study type	Sample size	Gender	Age (years)	Mechanism of dislocation	Risk factors	Definitive management	Outcome
Bonnaig (9)	Case report	1	Male	59	Spontaneous	Intraarticular injection, sarcoidosis, leukopenia	Scheduled for arthrodesis	NR
Oztürkmen (10)	Case report	1	Women	65	Spontaneous following minimal sprain	None	TKR	Pain free, with full ROM after 3 years

NR: not reported, TKR: total knee replacement, ROM: range of motion.

### Pathogenesis of septic arthritis

The etiology of septic arthritis in our patient was unknown with no history of possible inoculation such as history of steroid injection, IV drug abuse or recent knee surgery. However, it can be postulated that the etiology might be attributable to her past medical history of DM and CKD. In their systematic review, Margaretten *et al*. [1] showed that DM is an important risk factor when assessing the probability of septic arthritis in patients. Another reason could be the presence of CKD, in which a previous study showed that patient with end stage renal disease (ESRD) are at 50 folds increased risk of septic arthritis than the general population [2]. Although our patient did not have ESRD, her history of CKD makes her more susceptible to septic arthritis and eventually bacteremia and sepsis than the general population. These severe sequelae in patients with kidney disease is thought to be due to the uremia and chronic vascular access, which eventually leads to immune system dysfunction and increased risk of infections [3].

### Management of septic arthritis-related knee dislocation

One of the methods used in stabilizing and reducing knee dislocation is a spanning external fixator. In their study, Berliner *et al*. [4] used a 6-week period of spanning external fixator for a patient with traumatic knee dislocation and showed adequate stability for preventing further dislocations. However, the application of a spanning external fixator has failed in our case. This might be because of the non-traumatic nature of the dislocation, in which the primary cause of dislocation was septic arthritis that could have led to the destruction of ligaments and made it fragile to withstand weight-bearing. Hence, when knee dislocation happens as a consequence of septic arthritis, a definitive management should be pursued to prevent further damage and achieve the most suitable treatment goal and patient satisfaction.

The definitive management of such case can be challenging with different surgical options such as arthrodesis, resection arthroplasty with cement spacer and total knee replacement (TKR). In this report, several factors persuaded our decision of choosing arthrodesis over TKR. The patient recurrent history of septic arthritis was one factor. Another factor was the patient history of DM and CKD, which placed the patient under a risk of immune compromise and an increased risk of recurrence. Therefore, TKR possessed a greater risk of recurrent infection and further complications in our patient. Similarly, Bonnaig *et al*. [5] reportof a spontaneous knee dislocation after septic arthritis, the patient’s risk factors such as sarcoidosis and leukopenia made the patient in greater risk of total knee prosthesis infection, therefore, making arthrodesis more favorable in their case. However, when eradication of infection is possible and the patient possesses no risk factors that may increase the risk of prosthesis infection, TKR would be a suitable management option as demonstrated in Oztürkmen *et al*. [6] case report.

## Data Availability

The data that support the findings of this study are available from the corresponding author upon reasonable request.
